# Characteristics of eye-related emergency visits and triage differences by nurses and ophthalmologists: Perspective from a single eye center in southern China

**DOI:** 10.3389/fmed.2023.1091128

**Published:** 2023-03-16

**Authors:** Juan Chen, Chen-Mei Chen, Yongxin Zheng, Liuxueying Zhong

**Affiliations:** State Key Laboratory of Ophthalmology, Zhongshan Ophthalmic Center, Sun Yat-sen University, Guangdong Provincial Key Laboratory of Ophthalmology and Visual Science, Guangdong Provincial Clinical Research Center for Ocular Diseases, Guangzhou, Guangdong, China

**Keywords:** ophthalmic emergencies, emergency department, characteristics, triage, nurses

## Abstract

**Purpose:**

To describe characteristics of eye-related emergency department (ED) visits and investigate differences in priorities assigned to patients by triage nurses and ophthalmologists.

**Methods:**

A prospective survey was conducted at the ED of Zhongshan Ophthalmic Center from January 1, 2021, to May 31, 2021. Clinical data from patients with acute ophthalmic conditions lasting less than 7 days were collected *via* a standard questionnaire and the urgency levels assigned by nurses and physicians were also recorded. Binary logistic regression was performed to identify characteristics associated with truly emergency conditions and up- or down-triage.

**Results:**

A total of 1907 patients were enrolled, with 582 (30.5%) classified as “non-emergency.” Red eye (69.7%), eye pain (53.0%), ocular trauma (44.1%), tearing (43.6%), and blurred vision (43.1%) were the most common complaints. Truly emergency tended to be male (OR 2.019, *p* < 0.001) and with unilateral eye involvement (OR 2.992, *p* < 0.001). Nurses prioritized conjunctival, scleral, closed ocular trauma and eyelid diseases over doctors while giving less priority to open ocular trauma, cornea, uveitis, and vitreoretinal diseases (*p* < 0.05). Overemphasis on mild blurred vision (OR 3.718, *p* = 0.001) and insufficient understanding of conjunctival diseases without red eye (OR 0.254, *p* = 0.001) were associated with conjunctival disease “up-triage.” Insufficient awareness of moderate and severe blurred vision was associated with “down-triage” for ocular trauma (OR 3.475, *p* = 0.001 and OR 2.422, *p* = 0.020, respectively).

**Conclusion:**

Ophthalmic EDs are typically flooded with patients suffering from acute ocular problems, with a considerable portion for non-emergency conditions. The identification of characteristics associated with truly emergency cases and nurses’ triage preferences is valuable in providing target guidance for future ED practice and facilitating the proper allocation of emergency resources.

## Introduction

Ophthalmic emergencies are rapidly progressing vision-threatening disorders that must be treated as soon as possible to avoid permanent vision loss (1, 2). However, a variety of minor ailments mimicking urgent conditions, such as eye redness or eye pain, may bother patients enough to visit the emergency departments (EDs), causing crowding and endangering critically ill patients (3). As estimated in the United States from 2010 to 2017, 44.8% of 16.8 million eye-related emergency department visits were for non-emergent conditions (4). However, few studies have been conducted in China to assess the epidemiology of ocular emergencies. Nonetheless, data are scarce on the clinical characteristics of ophthalmic ED patients and risk factors for true emergencies. Identifying the information not only informs current resource allocation but also provides guidance for prior emergency healthcare reform.

Triage is an effective process for ensuring that effective emergency care is provided despite limited resources by sorting and prioritizing patients to appropriate orders for visits based on their urgency degree. Risk stratification is one of the central challenges of emergency medicine (5–7). Due to non-eye practitioners’ limited knowledge and training in ophthalmology, the problem of identifying vision-threatening patients is highlighted in ophthalmic emergencies (8, 9). Historically, registered nurses have been the first line to perform triage in many countries’ EDs (6). Previous studies have found that nurses in general EDs tend to assign patients to more urgent categories to avoid missing critical patients (10, 11). However, there are currently little data in the literature on nurses’ prioritization preference for ophthalmic emergencies. Besides, little is known about which symptoms are over-emphasized or ignored by nurses, resulting in up- or down-triage. Investigating how triage-related tasks are carried out would allow us to perform target training to improve the current ophthalmic triage quality.

Our study aims to describe eye-related ED visits from a perspective of a large eye center ED in southern China, compare the triage priority assigned to patients between nurses and ophthalmologists, and identify risk factors linked with true urgency and triage differences. A thorough understanding of ophthalmic EDs could identify the pressing situation, develop a predictive model, and enhance quality in future healthcare practices.

## Materials and methods

### Study design

This was a prospective cross-sectional study involving patients with acute eye-related conditions who visited Zhongshan Ophthalmic Center (ZOC). The study was approved by the Ethics Reviewer Board of ZOC (approval number: 2021KYPJ046). Standard medical procedures were not stopped during the research, and the study was conducted in accordance with the Declaration of Helsinki.

### Study population

The inclusion criteria were: (1) patients presenting to the ZOC emergency department for the first time with acute ophthalmic symptoms lasting less than 7 days between January 1, 2021, and May 31, 2021. (2) Complete records of symptoms, ocular trauma history, medical history, and final diagnosis. (3) Complete urgency levels labeled by both nurses and ophthalmologists. The exclusion criteria were: (1) patients who revisited as requested by a doctor. (2) Ocular symptoms that continued longer than 7 days. (3) Records with insufficient information (symptoms, ocular trauma history, medical history, final diagnosis, or urgency labels).

### Data collection and definition

During the study period, we designed a standardized questionnaire to collect patients’ medical data: demographics, ocular trauma history, presenting symptoms, and medical history. At the time of visits, the urgent levels labeled by the nurses and physicians were collected. In addition, we also recorded the final diagnosis of each patient.

A three-level triage classification system for ophthalmic emergencies was amended from the prior literature (1, 3, 12, 13). In brief, we classified ophthalmic emergencies into three levels based on the severity of the disease and the time window in which urgent care was required. The term “emergency” was defined as a condition requiring immediate treatment, where a delay would result in disastrous vision loss. For instance, open globe injuries, central retinal artery occlusion, acute glaucoma, etc. “Semi-emergency” referred to a condition that needed to be treated within 24 h and for which a delay would result in great discomfort or a less favorable visual outcome. For instance, rhegmatogenous retinal detachment, blunt ocular trauma, or corneal abrasion. “Non-emergency” was the ocular disorder that could be treated at outpatient clinics as opposed to emergency rooms, like conjunctivitis and subconjunctival hemorrhage.

### Statistical analysis

Statistical analyses were conducted using IBM SPSS, version 22. For frequency distributions, we calculated the proportion of positive cases to the total number of patients. Continuous variables were presented as the means with standard deviation (SD). Cohen’s Kappa was used to evaluate the triage agreement between nurses and physicians. After grouping the diagnoses, we further estimated whether nurses tended to give a higher or less priority to specific illnesses than doctors. The difference in triage disagreement assigned to the two categories (doctors > nurses or nurses > doctors) was compared using Pearson’s chi-square or Fisher’s exact test where the expected frequency in any cell was less than five. Binary logistic regression analysis was performed to identify characteristics associated with true emergency and triage discrepancy. Statistical significance was defined as a two-tailed *p* < 0.05.

## Results

### Demographics and presentation characteristics of the study population

Over the 5-month research period, a total of 1907 ED visits with acute ocular conditions were enrolled in our study (Table 1). Of these, 448 (23.5%) patients were classified as “emergency,” 877 (46.0%) as “semi-emergency,” and 582 (30.5%) as “non-emergency.” The mean age was 40 years (SD ± 17.5 years, ranging from 1 to 91 years) and males accounted for 61.1% (1,165 patients).

**Table 1:** Overall description of the study population.

	*n*	% (95CI)
**Triage level**		
*E*	448	23.5 (21.6–25.4)
Semi-*E*	877	46.0 (43.7–48.2)
Non-*E*	582	30.5 (28.5–32.6)
Gende*r*		
Male	1,165	61.1 (58.9–63.3)
Female	742	38.9 (36.7–41.1)
Age (years)		
Mean ± SD	40.0 ± 17.5	
**Reasons for consultation** ^a^		
Red eye	1,330	69.7 (67.7–71.8)
Eye pain	1,010	53.0 (50.7–55.2)
Ocular trauma	841	44.1 (41.9–46.3)
Tearing	832	43.6 (41.4–45.9)
Blurred vision	821	43.1 (40.8–45.3)
Photophobia	634	33.2 (31.1–35.4)
Increased eye secretion	207	10.9 (9.5–12.3)
Foreign body sensation	196	10.3 (8.9–11.6)
Itching eye	154	8.1 (6.9–9.3)
Floats	149	7.8 (6.6–9.0)
Visual field defect	135	7.1 (5.9–8.2)
Swollen and painful eyelid	95	5.0 (4.0–6.0)
Flashes	39	2.0 (1.4–2.7)
Others	162	8.5 (7.2–9.7)

^a^Patients can choose more than one answer.

We further summarized the presentation characteristics of patients visiting the ED (Table 1). The most common reasons for consultation were red eye (69.7%), eye pain (53.0%), ocular trauma (44.1%), tearing (43.6%), and blurred vision (43.1%).

### Disease spectrum of eye-related ED visits and risk factors for true urgency

**Figure 1:**
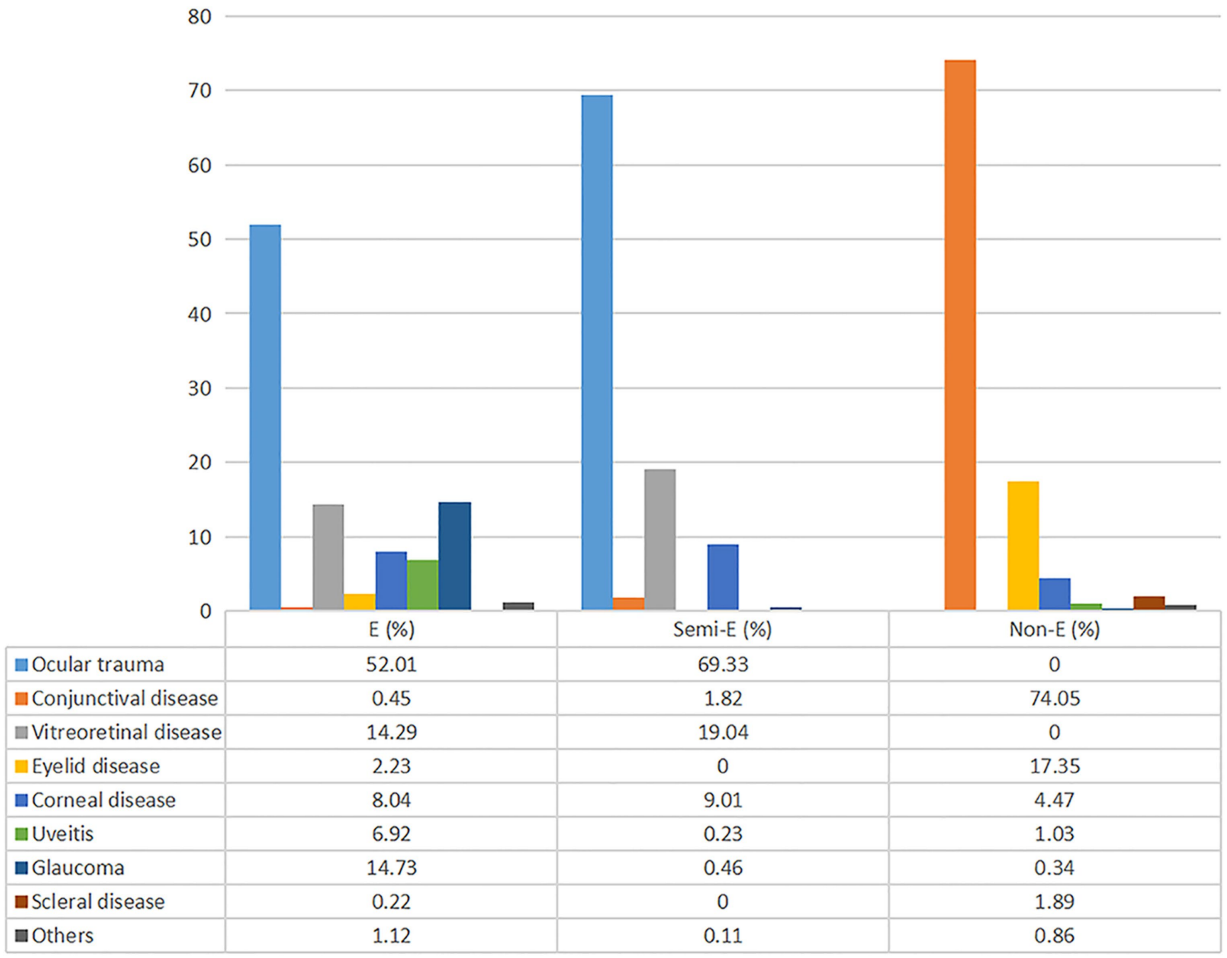
Disease spectrum of eye-related emergency visits based on different triage grades. E, emergency; Semi-E, semi-emergency; Non-E, non-emergency.

In general, ocular trauma (44.1%), conjunctival disease (23.5%), and vitreoretinal disease (12.1%) were the leading diagnosis for ophthalmic ED visits. Figure 1 showed the diagnostic constitution in each of the three urgency categories. For emergency and semi-emergency groups, ocular trauma (52.0 and 69.3%, respectively), glaucoma (14.7 and 0.5%, respectively), vitreoretinal disease (14.3 and 19.0%, respectively) and corneal disease (8.0 and 9.0%, respectively) were the most common diagnoses, while conjunctival (74.0%) and eyelid (17.3%) diseases occurred most frequently for the non-emergency group.

Binary logistic regression showed that male (OR 2.019, *p* < 0.001) and unilateral eye involvement (OR 2.992, *p* < 0.001) were risk factors for true urgency (including emergency and semi-emergency) (Table 2). Compared with non-emergency cases, truly urgent cases were more likely to be eye pain (OR 3.703, *p* < 0.001), ocular trauma (p < 0.001), tearing (OR 2.985, *p* < 0.001), blurred vision (OR 11.739, *p* < 0.001), photophobia (OR 4.844, *p* < 0.001), floats (*p* < 0.001), visual field defect (*p* < 0.001), flashed (*p* < 0.001), and less likely to be red eye (OR 0.603, *p* < 0.001), increased eye secretion (OR 0.164, *p* < 0.001), itching eye (OR 0.042, *p* < 0.001), swollen and painful eyelid (OR 0.044, *p* < 0.001).

**Table 2:** Binary logistic regression to assess characteristics associated with true urgency.

	Numbers		OR (95% CI)	*p*
	*E* + Semi-*E* (*n* = 1,325)	Non-*E* (*n* = 582)		
**Gender**				
Male	878	287	2.019 (1.656–2.462)	<0.001
Age (years)				
<20 (Ref)	177	72	1.0	–
20–29	221	89	1.010 (0.699–1.460)	0.957
30–39	263	121	0.884 (0.624–1.253)	0.489
40–49	241	107	0.916 (0.642–1.308)	0.630
50–59	258	108	0.972 (0.682–1.385)	0.874
60 or older	165	85	0.790 (0.541–1.153)	0.222
**Unilateral eye involvement**				
Yes	1,258	502	2.992 (2.128–4.207)	<0.001
**Symptoms**				
Red eye	883	447	0.603 (0.482–0.755)	<0.001
Eye pain	829	181	3.703 (3.008–4.558)	<0.001
Ocular trauma	841	0	^a^	<0.001
Tearing	680	152	2.985 (2.410–3.690)	<0.001
Blurred vision	761	60	11.739 (8.797–15.665)	<0.001
Photophobia	558	76	4.844 (3.717–6.329)	<0.001
Increased eye secretion	66	141	0.164 (0.120–0.224)	<0.001
Foreign body sensation	141	55	1.141 (0.120–0.224)	0.430
Itching eye	17	137	0.042 (0.025–0.071)	<0.001
Floats	149	0	^a^	<0.001
Visual field defect	135	0	^a^	<0.001
Swollen and painful eyelid	10	85	0.044 (0.023–0.086)	<0.001
Flashes	39	0	^a^	<0.001

^a^Because there was a frequency data of 0 in the cell, Fisher’s exact test was used. OR, odds ratio; Ref, reference.

### Comparison of the tendency to triage priority by nurses and by ophthalmologists

Table 3 displayed the overall assignment to different urgency categories as performed by triage nurses and ophthalmologists. Overall, there was relatively strong agreement between the two groups (Cohen’s Kappa = 0.715, 95%CI = 0.688–0.742). After further investigating the disagreement part between the two groups, we discovered that nurses had a tendency to grade patients as “semi-emergency” rather than “emergency” or “non-emergency” like doctors did.

**Table 3:** Triage distribution of 1907 patients by nurses and by ophthalmologists.

		Ophthalmologists			Total
		*E*	Semi-*E*	Non-*E*	
Nurses	*E*	284	39	1	324
	Semi-*E*	148	780	78	1,006
	Non-*E*	16	58	503	577
	Total	448	877	582	1907

Kappa = 0.715 (95% CI = 0.688–0.742), *p* < 0.001.

We classified patients based on their final diagnosis to see if there was a triage priority tendency for different diseases between nurses and ophthalmologists. Table 4 displayed the number of cases assigned to each type of diagnosis, as well as the numbers where doctors assigned a higher degree of urgency than nurses, the numbers where nurses assigned a higher degree, and the significance of the difference in proportions assigned to the two categories (“doctors > nurses” and “nurses > doctors”). Statistical results showed that the proportion of the “doctors > nurses” wing was significantly higher than the “nurses > doctors” wing for the triage of open ocular trauma (χ^2^ = 11.227, *p* = 0.001), cornea (χ^2^ = 7.257, *p* = 0.007), glaucoma (χ^2^ = 11.415, *p* ≤ 0.001), uveitis (χ^2^ = 6.139, *p* = 0.015), and vitreoretinal diseases (χ^2^ = 10.535, *p* = 0.001), indicating that nurses tended to give less priority to these diseases. Among them, nurses’ classification for open ocular trauma, glaucoma, and fundus diseases was particularly underestimated (χ^2^ > 10, *p* ≤ 0.001), suggesting their disability to distinguish diseases that could seriously damage vision from “semi-emergency” or “non-emergency” cases, resulting in “down-triage.” Furthermore, nurses rated patients with conjunctival lesions (χ^2^ = 73.272, *p* ≤ 0.001), sclera diseases (*p* = 0.041), closed ocular trauma (χ^2^ = 5.404, *p* = 0.020) and eyelid diseases (χ^2^ = 5.181, *p* = 0.023) as more urgent than doctors.

**Table 4:** Comparison of the tendency to up- or down-triage based on diagnosis.

Diagnosis	Total	Doctors > nurses	Agree	Nurse > doctors	χ^2^	*p*
Open ocular trauma	132	24	107	1	11.227	0.001^a^
Closed ocular trauma	624	32	563	29	5.404	0.020^a^
Other ocular trauma	85	16	67	2	4.669	0.031^a^
Cornea	141	33	102	6	7.257	0.007^a^
Conjunctiva	449	11	388	50	73.272	<0.001^a^
Sclera	12	0	9	3		0.041^b^
Glaucoma	72	28	42	2	11.415	<0.001^a^
Uveitis	39	22	14	3	6.139	0.013^a^
Vitreous and retina	231	42	182	7	10.535	0.001^a^
Eyelid	111	10	88	13	5.181	0.023^a^
Others	11	4	5	2		1.000^b^
Total	1907	222	1,567	118		

^a^Data comparison between two groups using chi-square test. ^b^Data comparison between two groups using Fisher’s exact test.

### Risk factors for up- or down-triage of nurses

Binary logistic regression analysis was conducted to identify risk factors for up- or down-triage in a specific disease (Table 5). For conjunctival disease, univariate analyses indicated that red eye, intolerable eye pain, and mild blurred vision were associated with up-triage. The multivariate analyses showed that mild blurred vision (OR 3.718, *p* = 0.001) and red eye (OR 0.254, *p* < 0.001) were independent risk factors for up-triage. Similarly, for ocular trauma, univariate analyses revealed that moderate and severe blurred vision, tearing, photophobia, and floats were associated with down-triage. The multivariate analyses showed that moderate and severe blurred vision (OR 3.475, *p* < 0.001, and OR 2.422, *p* = 0.020) were independent risk factors for down-triage.

**Table 5:** Binary logistic regression analysis to assess risk factors for conjunctival disease up-triage and ocular trauma down-triage.

	Conjunctival disease					Ocular trauma				
	Up-triage/agree	Univariate		Multivariate		Down-triage/agree	Univariate		Multivariate	
		OR	*p*	OR	*p*		OR	*p*	OR	*p*
Red eye (yes/no)	37/354	0.273	**<0.001**	0.254	**<0.001**	58/594	1.221	0.520		
Eye pain										
None (Ref)	28/273	1	–	1	–	21/240	1	–		
Tolerable	22/110	1.950^a^	**0.029**	1.767	0.074	38/421	1.032	0.913		
Intolerable^b^	0/5		1.000			13/108	1.376	0.391		
Blurred vision										
None (Ref)	39/358	1	–	1	–	20/384	1	–	1	–
Mild	11/28	3.606^a^	**0.001**	3.718	**0.001**	9/170	1.016	0.968	0.876	0.751
Moderate^c^	0/2		1.000			30/126	4.571	**<0.001**	3.475	**<0.001**
Severe^d^	0/0	–	–			13/89	2.804	**0.006**	2.422	**0.020**
Tearing (yes/no)	15/103	1.186	0.605			53/435	2.142	**0.006**	1.689	0.182
Photophobia (yes/no)	8/43	1.528	0.311			43/353	1.747	**0.026**	1.121	0.748
Increased eye secretion (yes/no)	14/99	1.135	0.706			5/27	2.051	0.154		
Foreign body sensation (yes/no)	7/41	1.378	0.466			11/112	1.058	0.870		
Itching eye (yes/no)	15/93	1.359	0.353			2/5	4.366	0.081		
Floats (yes/no)	0/0	–	–			9/31	3.401	**0.002**	2.176	0.074
Visual field defect (yes/no)	0/0	–	–			1/9	1.189	0.870		

^a^Because there was a frequency data of 0 in the cell, Fisher’s exact test was used. ^b^Intolerable, degree of need for painkillers; ^c^Moderate, inability to recognize a face 6 feet away; ^d^inability to recognize his own outstretched fingers. OR, odds ratio; Ref, reference. Bold values mean statistically significant differences (*p* < 0.05).

## Discussion

Ophthalmic emergencies account for a small but significant proportion of general hospitals’ EDs or the ophthalmic EDs of dedicated eye centers. Although a single ophthalmic emergency is rarely life-threatening, delayed treatment may lead to severe vision loss and subsequent poor quality of life. Prior ED studies focused on either general emergency medicine (14, 15) or ocular trauma emergencies (16–18), and they were mainly conducted in the United States and other developed countries, with little information from developing countries (4, 18–20). To our knowledge, no researches have been done on ophthalmic nurses’ preferences for triage and associated risk factors. Our study provided useful information on the epidemiology of ophthalmic crises (both traumatic and non-traumatic) and the quality of nurse triage from a perspective of a large eye center in Southern China.

As initially expected, we confirmed that a sizable portion (30.5%) of all eye-related ED visits were for non-emergent conditions. Compared to the 44.8% reported in the United States (4), the lower proportion of non-urgent cases in our study may be attributable to the fact that our study took place in a specialized eye hospital where triage nurses with extensive ophthalmic experience assigned non-urgent patients and suggested appointments to outpatient clinics. Ocular lesions were easily recognized because the eyes were superficial and our most significant sense organ. Patients may go to EDs for minor ailments that mimic urgent concerns, such as eye pain or redness. According to a survey from the Nationwide Emergency Department Sample (NEDS) database, more than 4 million eye-related ED visits were for conjunctivitis (28.0%), subconjunctival hemorrhages (3.0%), and styes (3.8%) (19). Likewise, our study found that conjunctival (74.0%) and eyelid (17.3%) diseases were the leading diagnoses for non-emergency visits. Therefore, increasing public awareness and education about common ocular “acute” disorders, as well as transferring these visits to appointment clinics, were promising strategies for improving the allocation of emergency medical resources.

According to our research, red eyes, eye pain, ocular trauma, tearing, and blurred vision accounted for more than 90% of visits to ophthalmic EDs. Our study had a greater percentage of ocular trauma than surveys by Agrinier et al. and Suzie Kim et al. (44.1% versus 26.1 and 31.6%) (21, 22). The high percentage of eye injuries may be related to China’s social and economic structure since there were many people employed in the agricultural and industrial sectors, which increased the risk of ocular trauma.

Because the ophthalmic EDs were scattered with patients suffering various acute symptoms, it was crucial to identify the traits of true urgency. Typically, nurses or general practitioners relied on subjective judgment to assess patients, with high variation (8, 23). In order to address subjective disparities in human triage and improve the efficiency of EDs, some scholars began to design triage scales suitable for ophthalmic emergencies (24–26). By retrospective analysis of electronic medical records and clinical experience, they selected simple-to-assess symptoms for ophthalmic triage, such as “redness,” “pain,” “loss of vision,” and “eye trauma,” and then assigned different scores to each symptom. The final emergency level was then determined from the total score. The researchers, however, did not provide explanations or principles for the scores assigned to different symptoms. Our study prospectively collected the symptoms from the patients’ perspective and calculated the ORs of different characteristics for emergency classifications, which will provide a theoretical basis for the selection and scoring principles of triage characteristics in the future, whether it is used to design a new triage scale or an artificial intelligence prediction tool.

Previous studies on EDs in general hospitals revealed that there was poor agreement in triage between nurses and physicians, and nurses had a tendency to assign patients higher triage grades than physicians (10, 11), yet there was reasonably substantial agreement between the two groups in our records. There were two explanations for the distinction. First, ophthalmic crises were concentrated on ocular disorders and had fewer considerations than general ED visits, which must take into account systemic manifestations. Second, most general EDs employed a four- or five-level classification system (27–29), which made triage more challenging for nurses than the three-level triage in our study.

Interestingly, although we confirmed that there was strong agreement between nurses and doctors, we discovered that nurses tended to give a “semi-emergency” grade to all patients. So, we further evaluated whether nurses had a tendency to “up-triage” or “down-triage” for different types of diseases. Ocular trauma accounted for 44.1% of ophthalmic emergencies in our study. However, the fact that failure to recognize urgent ocular injury in our study was potentially serious, especially in those with open globe injuries. The reason for the “down-triage” was that the condition of ocular trauma was complex, and it was difficult for nurses to integrate all of the symptoms in a short period of time to acquire an accurate assessment comprehensively. On the contrary, it appeared that nurses placed too much priority on conjunctival diseases. This could be due to differences in doctors’ and nurses’ perceptions of ocular emergencies. Furthermore, the anxiety and high demand for ED visits exacerbated by patients with sudden ocular conditions played an important role in the up-triage.

One striking feature of our study was the exploration of risk factors for triage discrepancies, which could be used as the target training for nurses in future practice. Ocular trauma and conjunctival disease were the leading causes of eye-related ED visits, down-triage of ocular trauma may endanger patients’ vision, while up-triage of conjunctival disease was a waste of emergency department resources. Therefore, we investigated the risk factors for the mis-triage of these two diseases. According to statistical findings, mild blurred vision and a lack of eye redness were independent risk variables for conjunctival illnesses up-triage. So, we should focus on the education of relevant symptoms while training nurses. For instance, when a patient did not have red eye and only complain of a little decrease in vision, it did not indicate an urgent issue such as dry eye or asthenopia. While in the triage training of patients with ocular trauma, we should pay attention to assessing the degree of vision loss. Moderate and severe vision loss was a signal of urgency.

Our study still had several limitations. First, the study population source was limited to a single specialized eye hospital in southern China, with regional and disease severity differences that may not be generalizable to other settings. Second, because the population data was confined and the collection period was short without reflecting seasonal variations, additional studies with larger sample sizes and longer periods were considered.

## Conclusion

Our research revealed that a sizeable portion of ophthalmic ED visits was due to non-emergency conditions. The most common ophthalmic emergency complaints were red eye, eye pain, ocular trauma, tearing, and vision loss. Truly urgent cases were more likely to be male, unilateral eye involvement, eye pain, ocular trauma, tearing, blurred vision, photophobia, floats, visual field defect, flashed, and less likely to be red eye, increased eye secretion, itching eye, swollen and painful eyelid. When triaging, nurses tended to assign patients a “medium” grade and had different priority preferences for different kinds of diseases. The risk factors that led to differences in triage between doctors and nurses varied depending on diseases. Therefore, we should strengthen targeted training to increase nurses’ subjective priority preference and increase the objectivity of triage decisions.

## Data availability statement

The original contributions presented in the study are included in the article/supplementary material, further inquiries can be directed to the corresponding authors.

## Ethics statement

The studies involving human participants were reviewed and approved by the Ethics Committee of the Zhongshan Ophthalmic Center, Sun Yat-Sen University, Guangzhou, Guangdong (2021KYPJ046). Written informed consent to participate in this study was provided by the participants’ legal guardian/next of kin.

## Author contributions

LZ and YZ: conceptualization and resources. JC and C-MC: methodology, software, and writing—original draft preparation. JC: formal analysis, investigation. LZ: writing—review and editing. YZ: project administration and funding acquisition. All authors contributed to the article and approved the submitted version.

## Funding

This study was supported by the Five-five Clinical Specialty Construction Project (3030901010068) and High-level Hospital Construction Project (303010402).

## Conflict of interest

The authors declare that the research was conducted in the absence of any commercial or financial relationships that could be construed as a potential conflict of interest.

## Publisher’s note

All claims expressed in this article are solely those of the authors and do not necessarily represent those of their affiliated organizations, or those of the publisher, the editors and the reviewers. Any product that may be evaluated in this article, or claim that may be made by its manufacturer, is not guaranteed or endorsed by the publisher.
