# Corrigendum: Characteristics of eye-related emergency visits and triage differences by nurses and ophthalmologists: perspective from a single eye center in southern China

**DOI:** 10.3389/fmed.2023.1241114

**Published:** 2023-07-12

**Authors:** Juan Chen, Chen-Mei Chen, Yongxin Zheng, Liuxueying Zhong

**Affiliations:** State Key Laboratory of Ophthalmology, Zhongshan Ophthalmic Center, Sun Yat-sen University, Guangdong Provincial Key Laboratory of Ophthalmology and Visual Science, Guangdong Provincial Clinical Research Center for Ocular Diseases, Guangzhou, Guangdong, China

**Keywords:** ophthalmic emergencies, emergency department, characteristics, triage, nurses

In the published article, there was an error in the affiliations for Chen-Mei Chen. Chen-Mei Chen should only have affiliation 1. Affiliation 2 “^2^Guangzhou Aier Eye Hospital, Guangzhou, Guangdong, China” should be removed.

The authors apologize for this error and state that this does not change the scientific conclusions of the article in any way. The original article has been updated.

